# Early Secondary Hyperparathyroidism and the Evolving Spectrum of Mineral and Bone Disorder in Chronic Kidney Disease: A Single-Centre Experience From Central India

**DOI:** 10.7759/cureus.113700

**Published:** 2026-07-30

**Authors:** Pranjal Kashiv, Amit S Pasari, Manish Balwani, Vivek Kute

**Affiliations:** 1 Nephrology, All India Institute of Medical Sciences, Nagpur, Nagpur, IND; 2 Nephrology, Saraswati Kidney Care Center, Nagpur, IND; 3 Nephrology, Jawaharlal Nehru Medical College, Wardha, IND; 4 Nephrology, Saraswati Kidney care Center, Nagpur, IND; 5 Nephrology, Institute of Kidney Diseases and Research Centre (IKDRC), Ahmedabad, IND

**Keywords:** chronic kidney disease, hyperphosphataemia, mineral and bone disorder, secondary hyperparathyroidism, vascular calcification, vitamin d deficiency

## Abstract

Background: Chronic kidney disease-mineral and bone disorder (CKD-MBD) is characterised by disturbances of calcium, phosphate and parathyroid hormone metabolism, and may be accompanied by vascular and valvular calcification. We set out to characterise stage-associated differences in these abnormalities in a Central Indian cohort, a setting for which region-specific data remain limited.

Methodology: In this retrospective, cross-sectional single-centre study, we reviewed the records of 150 consecutive patients with CKD stages 3-5D managed at a tertiary care centre in Central India between May 2024 and April 2025. Demographic, clinical and biochemical variables - albumin-corrected calcium, phosphorus, alkaline phosphatase (ALP), intact parathyroid hormone (iPTH) and 25-hydroxyvitamin D - were extracted, together with radiological assessment of vascular calcification by lateral abdominal radiography and of valvular calcification by echocardiography in patients with complete imaging records (*n* = 57). The stage-wise prevalence of each abnormality was determined and its relationship with calcification examined.

Results: Secondary hyperparathyroidism was the earliest and most consistent abnormality, present in 11 (45.8%) of stage 3 patients and rising to 50 (90.9%) by stage 5. Among dialysis-dependent (stage 5D) patients, the pattern broadened, with 11 (44.0%) showing elevated iPTH and 9 (36.0%) showing suppressed iPTH, suggestive of low bone turnover. Hypocalcaemia and hyperphosphataemia were uncommon in stages 3-4 but affected 25 (45.5%) and 33 (60.0%) patients, respectively, at stage 5 and 14 (56.0%) and 19 (76.0%) patients, respectively, at stage 5D, while the rise in ALP paralleled that of iPTH, consistent with increased bone turnover. Vitamin D deficiency was highly prevalent throughout (58%-80%) and showed no clear stage gradient. Vascular calcification increased from none in stage 3 to 5 (55.6%) in stage 5D, and valvular calcification from none to 2 (22.2%). Calcification was associated with higher phosphorus and iPTH and lower corrected calcium, but not with vitamin D status.

Conclusions: In this Central Indian cohort, CKD-MBD showed a coherent stage-associated pattern: early secondary hyperparathyroidism from stage 3, more frequent mineral derangement at advanced stages, and vascular and valvular calcification in advanced disease. While this pattern mirrors international observations, the high background burden of vitamin D deficiency and the frequency of late presentation lend a distinct regional character to the disorder. The findings support early and serial biochemical monitoring from stage 3, with stage-specific, individualised intervention aimed at limiting cardiovascular risk.

## Introduction

Chronic kidney disease (CKD) is a major public-health problem, affecting an estimated 5%-10% of the global population [1]. As kidney function declines, mineral metabolism is progressively disturbed: the serum and tissue concentrations of calcium and phosphorus are disrupted, and the circulating hormones that regulate them, namely parathyroid hormone (PTH), vitamin D metabolites and fibroblast growth factor-23 (FGF-23), are altered [2]. As early as stage 3, the kidney's capacity to excrete phosphate is impaired, driving hyperphosphataemia, reduced calcitriol synthesis, secondary hyperparathyroidism and elevated FGF-23 [3]. Resistance to the actions of both PTH and vitamin D at the tissue level develops in parallel, further aggravating these abnormalities [4].

These interrelated derangements are grouped under the umbrella of CKD-mineral and bone disorder (CKD-MBD) [2]. The syndrome spans three domains: (1) abnormalities of calcium, phosphorus, PTH or vitamin D metabolism; (2) abnormalities of bone turnover, mineralisation or volume; and (3) vascular or soft-tissue calcification [5]. Each domain contributes materially to morbidity and mortality in CKD [6]. Crucially, cardiovascular disease, already the leading cause of death in this population, is closely linked to disturbed mineral metabolism, with vascular calcification emerging as a central pathological process [7].

The earliest and most consistent abnormality within this spectrum is secondary hyperparathyroidism (SHPT), which develops in a substantial proportion of patients by stage 3 and becomes almost universal as renal function declines further [3,5]. Alongside PTH elevation, hypocalcaemia, hyperphosphataemia, reduced 25-hydroxy and 1,25-dihydroxy vitamin D levels, and raised alkaline phosphatase (ALP) are frequently observed [8]. As disease advances into dialysis dependence, these derangements are compounded by extraskeletal calcification affecting both vascular and valvular structures, thereby accelerating cardiovascular risk [7,9].

The international framework for defining and managing CKD-MBD was refined in the KDIGO 2017 Clinical Practice Guideline Update [10]. The guideline emphasises serial rather than isolated assessment, with monitoring of serum calcium, phosphate and PTH beginning at CKD stage G3a. Suggested intervals for calcium and phosphate are every 6-12 months in G3a-G3b, every 3-6 months in G4, and every 1-3 months in G5/G5D; PTH is measured every 6-12 months in G4 and every 3-6 months in G5/G5D. ALP should be assessed at least annually in advanced CKD, or more frequently when PTH is elevated [10]. Vitamin D deficiency or insufficiency should be corrected using general-population strategies, and measurement of 25-hydroxyvitamin D (25(OH)D) is suggested across G3a-G5D [11].

Therapeutic recommendations have likewise shifted. In G3a-G5D, phosphate-lowering therapy is reserved for progressively or persistently elevated phosphate [10]. Hypercalcaemia should be avoided, and mild, asymptomatic hypocalcaemia may be tolerated in adults to limit calcium loading [6,10]. For PTH, no single optimal value is defined in non-dialysis CKD, but persistently or progressively rising levels above the assay's upper limit should prompt evaluation of modifiable factors [5]. In dialysis-dependent patients, KDIGO suggests maintaining PTH within two to nine times the assay's upper normal limit, using calcimimetics, calcitriol or vitamin D analogues, alone or in combination [10]. Together, these updates mark a contemporary move away from rigid numeric targets towards individualised, trend-based decision-making.

In India, additional challenges apply. Vitamin D deficiency is widespread in the general population, nutritional patterns vary, and access to early nephrology care remains limited. As a consequence, patients frequently present in advanced CKD, when biochemical abnormalities are already severe and complications established. These regional features may alter the onset, severity and pattern of CKD-MBD relative to Western cohorts, underscoring the need for local data [12,13].

Against this background, we undertook a retrospective, cross-sectional single-centre study in Central India to characterise the prevalence and pattern of MBD across CKD stages 3-5D. The specific objectives were to: determine the prevalence of CKD-MBD abnormalities at each CKD stage; characterise the associated biochemical abnormalities, namely calcium, phosphorus, PTH, ALP and vitamin D; and assess the presence of vascular and valvular calcification and its relationship to CKD stage.

By systematically integrating biochemical profiles with radiological evidence in a Central Indian cohort, this study aims to provide region-specific insight into the pattern of CKD-MBD and thereby to inform earlier recognition and more targeted intervention.

## Materials and methods

Study design and setting

We undertook a retrospective, cross-sectional, single-centre observational study at a tertiary care hospital in Central India, reviewing the records of all patients with CKD stages 3 to 5D who attended the nephrology service between May 2024 and April 2025.

Study population

Eligibility was restricted to adults aged 18 years or older with CKD stage 3, 4, 5 or dialysis-dependent stage 5D, staged in accordance with KDIGO criteria, for whom complete clinical and biochemical records were available. CKD was defined by abnormalities of renal structure or function persisting beyond three months, and the estimated glomerular filtration (eGFR) rate was calculated using the Modification of Diet in Renal Disease (MDRD) equation. Patients who had undergone prior renal transplantation or parathyroidectomy, and those with coexisting malignancy, advanced liver disease or active infection, were excluded. Given the retrospective and descriptive nature of the work, an a priori sample-size calculation was neither necessary nor appropriate; instead, every patient fulfilling these criteria during the study interval was enrolled consecutively, so that the resulting series of 150 constituted the entire eligible population, minimising selection bias in the biochemical cohort. Radiological assessment, however, was available only in a subset (*n* = 57), which was not systematically ascertained.

Ethical considerations

The study protocol received approval from the Institutional Ethics Committee. As the work comprised a retrospective review of pre-existing records, the requirement for individual consent was waived, and all data were anonymised before analysis.

Data collection

For each patient, we abstracted demographic information (age and sex) and the major comorbidities of hypertension, diabetes mellitus and cardiovascular disease, together with any documented clinical features of mineral and bone disorder, including bone pain, proximal myopathy, pruritus and skeletal deformity. The biochemical evaluation encompassed albumin-corrected serum calcium, serum phosphorus, ALP, intact parathyroid hormone (iPTH) and 25(OH)D; iPTH was assayed by electrochemiluminescence immunoassay, and 25(OH)D was interpreted against the laboratory's reference standards. Radiological assessment comprised lateral abdominal radiography for vascular calcification, hand radiography for skeletal manifestations such as subperiosteal resorption, and echocardiography for valvular calcification. Because such imaging is borne as out-of-pocket expenditure in our low- and middle-income setting, it could not be secured for every patient; calcification was therefore evaluated in the 57 individuals in whom imaging had been performed.

Definitions

Secondary hyperparathyroidism was defined as a serum iPTH exceeding the upper reference limit, whether or not accompanied by other biochemical abnormalities; suppressed iPTH was defined as a value below the lower reference limit, and elevated ALP as a value above the upper reference limit of the assay. Vitamin D deficiency denoted a 25(OH)D concentration below 20 ng/mL. Hyperphosphataemia was taken as serum phosphorus above 5.5 mg/dL in dialysis-dependent patients and above 4.5 mg/dL in those not receiving dialysis, and hypocalcaemia as an albumin-corrected calcium below 8.5 mg/dL. These thresholds conform to established criteria [3-10].

Statistical analysis

All analyses were performed in SPSS version 17.0 (SPSS Inc., Chicago, IL). Continuous variables were summarised as mean ± standard deviation or as median with interquartile range according to their distribution, and categorical variables as frequencies and percentages accompanied by Wilson 95% confidence intervals. The prevalence of each abnormality across CKD stages was compared using the Pearson chi-square test, with Cramér's V reported as the corresponding effect size, while graded relationships across the ordered CKD stages were assessed using the linear-by-linear (Cochran-Armitage) test for trend. In the calcification analyses, expected cell counts fell below five in several stage-specific cells, and the test for trend was therefore regarded as the primary comparison. Continuous variables were compared using the Student's t-test or one-way analysis of variance, and the distribution of calcification was examined according to CKD stage. Degrees of freedom accompanied each test statistic, and statistical significance was set at a two-sided *P*-value of < 0.05. Analyses were performed on available cases; no imputation was undertaken for missing data.

## Results

Baseline characteristics

The cohort comprised 150 patients with CKD stages 3 to 5D. Men predominated, numbering 100 (66.7%), at a male-to-female ratio of 2:1, and the mean age was approximately 50 years. The leading comorbidities were hypertension, present in 122 (81%), and diabetes mellitus, present in 68 (45%), while cardiovascular disease was documented in 15 to 21 patients (approximately 10 to 14%). Clinical features of mineral and bone disorder, namely bone pain, proximal myopathy and pruritus, were encountered more often at advanced stages (Table 1). Across the cohort as a whole, elevated iPTH was present in 104 patients (69.3%), vitamin D deficiency in 110 (73.3%), hyperphosphataemia in 68 (45.3%), hypocalcaemia in 58 (38.7%), elevated ALP in 41 (27.3%) and suppressed iPTH in 11 (7.3%).

**Table 1:** Baseline demographic and clinical profile (n = 150).

Parameter	Value
Mean age	~50 years
Sex, *n* (%)	Male 100 (66.7); female 50 (33.3)
Hypertension, *n* (%)	122 (81)
Diabetes mellitus, *n* (%)	68 (45)
Cardiovascular disease, *n* (%)	15 to 21 (≈10 to 14)
Common symptoms	Bone pain, proximal myopathy, pruritus

Stage-wise biochemical abnormalities

**Figure 1:**
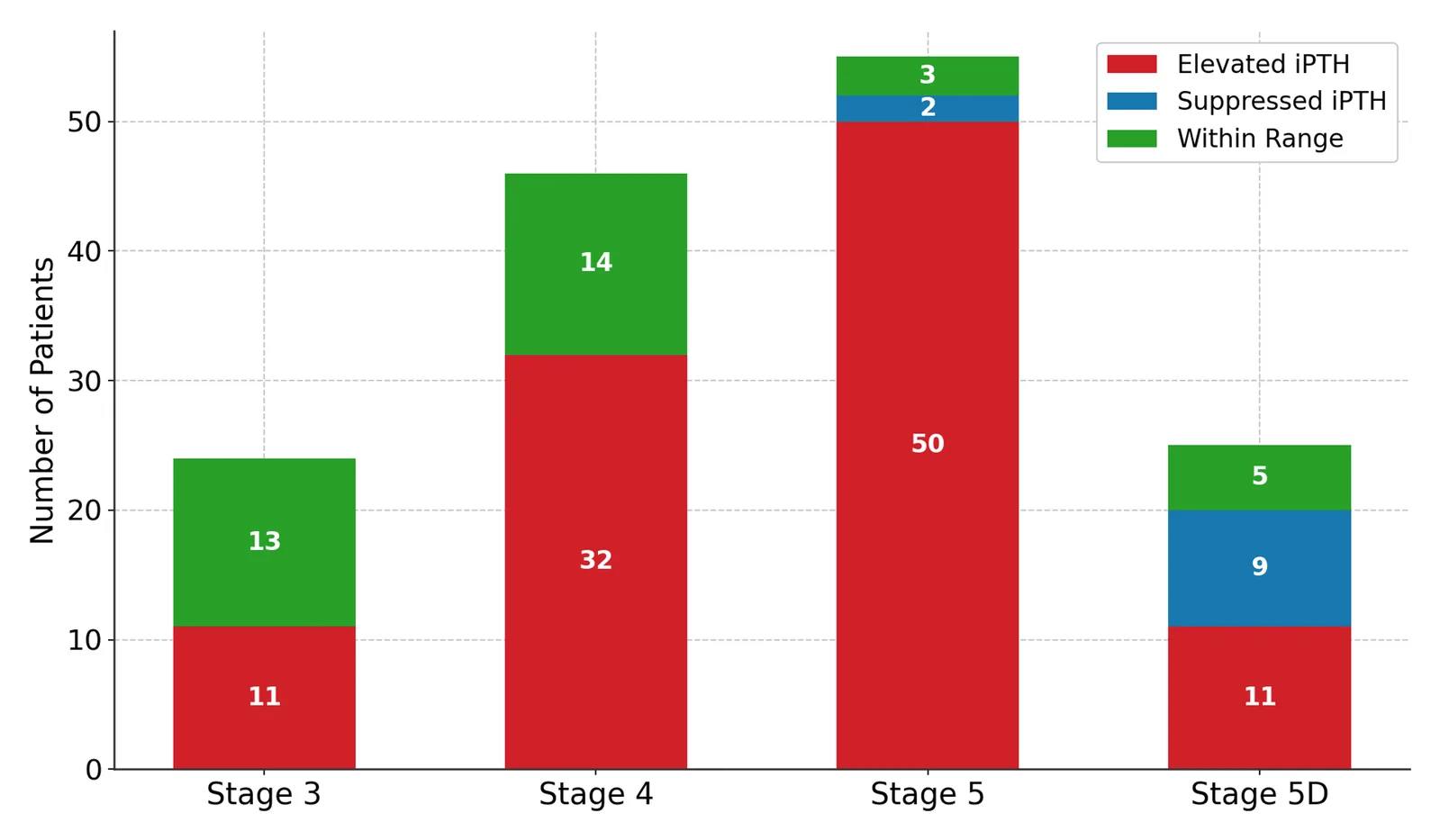
Distribution of intact parathyroid hormone (iPTH) status across CKD stages 3 to 5D. Stacked bars show the number of patients with elevated, suppressed, or within-range iPTH at each stage. Secondary hyperparathyroidism is evident from stage 3 and peaks at stage 5, while a suppressed-iPTH subgroup consistent with adynamic bone disease emerges at stage 5D. CKD, chronic kidney disease

Secondary hyperparathyroidism was the earliest and most consistent derangement. Elevated iPTH was identified in 11 (45.8%; 95% confidence interval (CI) 27.9-64.9) of stage 3 patients, rising to 32 (69.6%; 95% CI 55.2-80.9) at stage 4 and 50 (90.9%; 95% CI 80.4-96.1) at stage 5, a highly significant difference across stages (χ² = 25.82, df = 3, *P* < 0.001; Cramér's *V* = 0.41). At stage 5D, the picture diverged, 11 (44.0%) retaining elevated iPTH, 9 (36.0%) showing suppressed iPTH, suggestive of low bone turnover, and 5 (20.0%) lying within the reference range. This fall at stage 5D left the overall linear trend non-significant (trend χ² = 0.81, degrees of freedom (df) = 1, *P* = 0.37) despite the significant omnibus difference, a configuration that may reflect therapeutic modulation on dialysis and the emergence of low bone turnover, although treatment data were not available to test this (Figure 1; Table 2).

**Table 2:** Stage-wise biochemical abnormalities, n (%). Between-stage comparison (Pearson χ², degrees of freedom (df) = 3): elevated iPTH χ² = 25.82, *P* < 0.001, *V* = 0.41; hyperphosphataemia χ² = 30.11, *P* < 0.001, *V* = 0.45; elevated ALP χ² = 30.09, *P* < 0.001, *V* = 0.45; hypocalcaemia χ² = 8.77, *P* = 0.033, *V* = 0.24; low vitamin D χ² = 4.91, *P* = 0.18, *V* = 0.18. Linear-by-linear trend (df = 1): hyperphosphataemia and ALP *P* < 0.001; hypocalcaemia *P* = 0.003; low vitamin D *P* = 0.040; elevated iPTH *P* = 0.37 (non-monotonic, reflecting the stage 5D decline). ALP, alkaline phosphatase; iPTH, intact parathyroid hormone

Stage	*n*	Hypocalcaemia	Hyperphosphataemia	Elevated iPTH	Suppressed iPTH	Elevated ALP	Low vitamin D
3	24	5 (20.8)	3 (12.5)	11 (45.8)	0 (0.0)	0 (0.0)	14 (58.3)
4	46	14 (30.4)	13 (28.3)	32 (69.6)	0 (0.0)	5 (10.9)	32 (69.6)
5	55	25 (45.5)	33 (60.0)	50 (90.9)	2 (3.6)	22 (40.0)	44 (80.0)
5D	25	14 (56.0)	19 (76.0)	11 (44.0)	9 (36.0)	14 (56.0)	20 (80.0)

**Figure 2:**
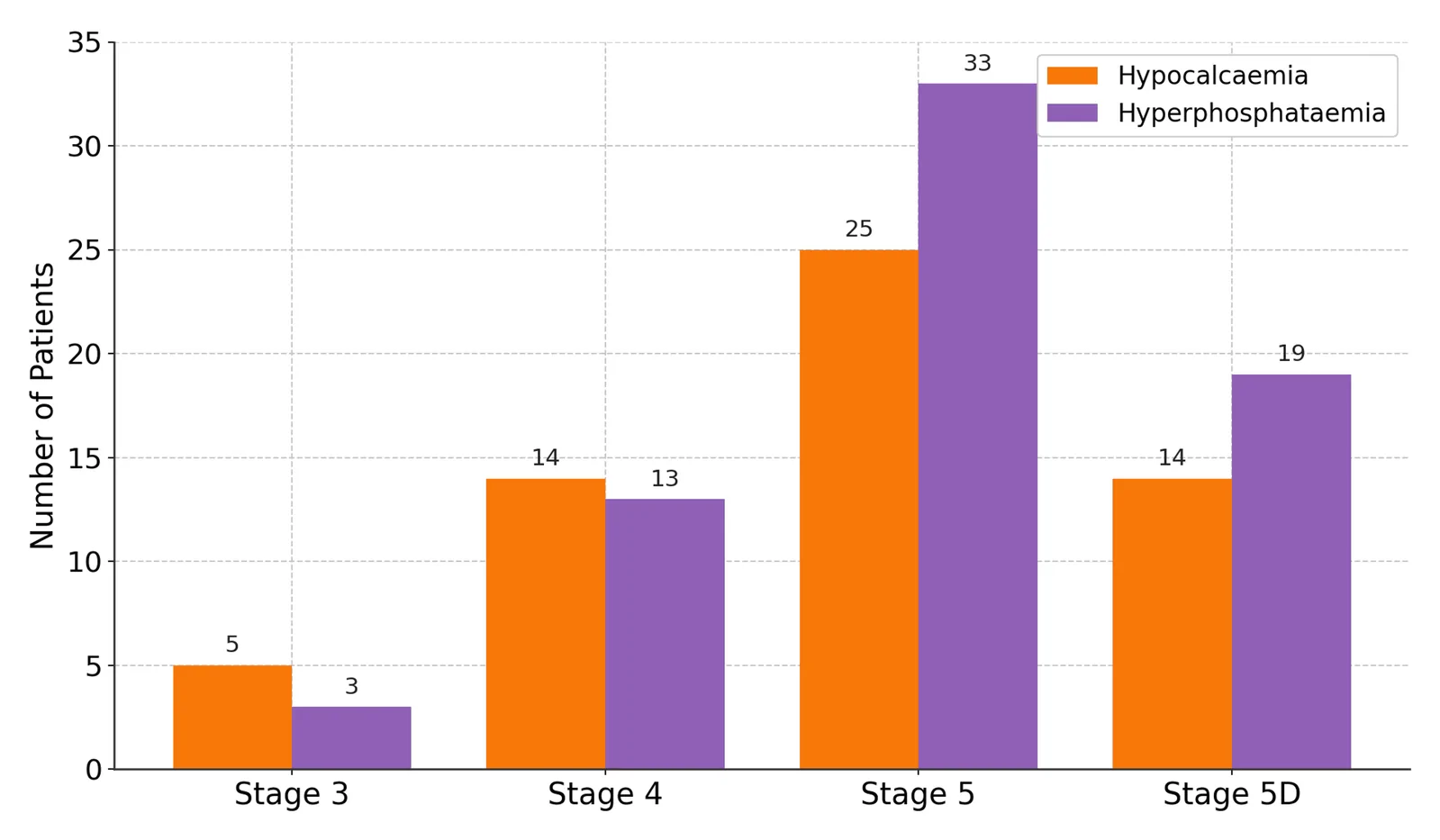
Stage-wise prevalence of hypocalcaemia and hyperphosphataemia. Both abnormalities were infrequent in stages 3 and 4 but increased sharply in stages 5 and 5D.

**Figure 3:**
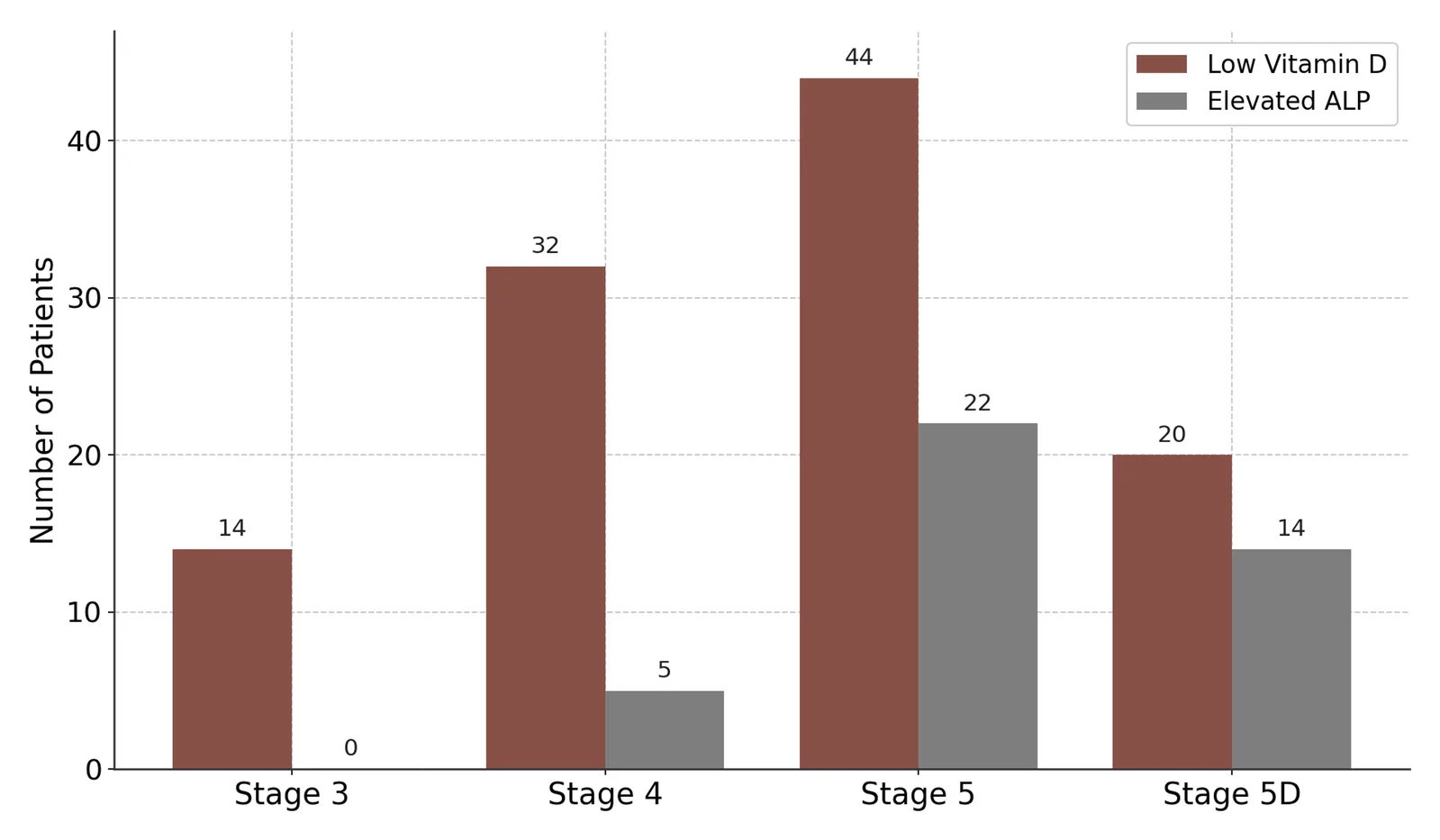
Stage-wise prevalence of vitamin D deficiency and elevated alkaline phosphatase (ALP). Vitamin D deficiency was highly prevalent at every stage, with no clear gradient, whereas ALP elevation increased progressively with advancing disease.

Disturbances of calcium and phosphate were uncommon in early disease but came to dominate the advanced stages. Hypocalcaemia grew progressively more frequent, occurring in 5 (20.8%), 14 (30.4%), 25 (45.5%; 95% CI 33.0-58.5) and 14 (56.0%; 95% CI 37.1-73.3) of patients in stages 3, 4, 5 and 5D, respectively (χ² = 8.77, df = 3, *P* = 0.033; *V* = 0.24; trend χ² = 8.62, df = 1, *P *= 0.003). Hyperphosphataemia rose more steeply still, from 3 (12.5%) at stage 3 to 19 (76.0%; 95% CI 56.6-88.5) at stage 5D (χ² = 30.11, df = 3, *P* < 0.001; *V* = 0.45; trend χ² = 28.99, df = 1, *P* < 0.001) (Figure 2). Alkaline phosphatase elevation followed the same course, increasing from 0 (0.0%) to 5 (10.9%) and 22 (40.0%) to 14 (56.0%) across the four stages (χ² = 30.09, df = 3, *P* < 0.001; *V* = 0.45; trend χ² = 28.70, df = 1, *P* < 0.001), in keeping with increased bone turnover. Vitamin D deficiency, by contrast, remained uniformly prevalent, affecting 14 (58.3%), 32 (69.6%), 44 (80.0%) and 20 (80.0%) of patients, with no significant difference between stages (χ² = 4.91, df = 3, *P* = 0.18) and only a weak upward trend (trend χ² = 4.22, df = 1, *P* = 0.040) (Figure 3).

Vascular and valvular calcification

**Figure 4:**
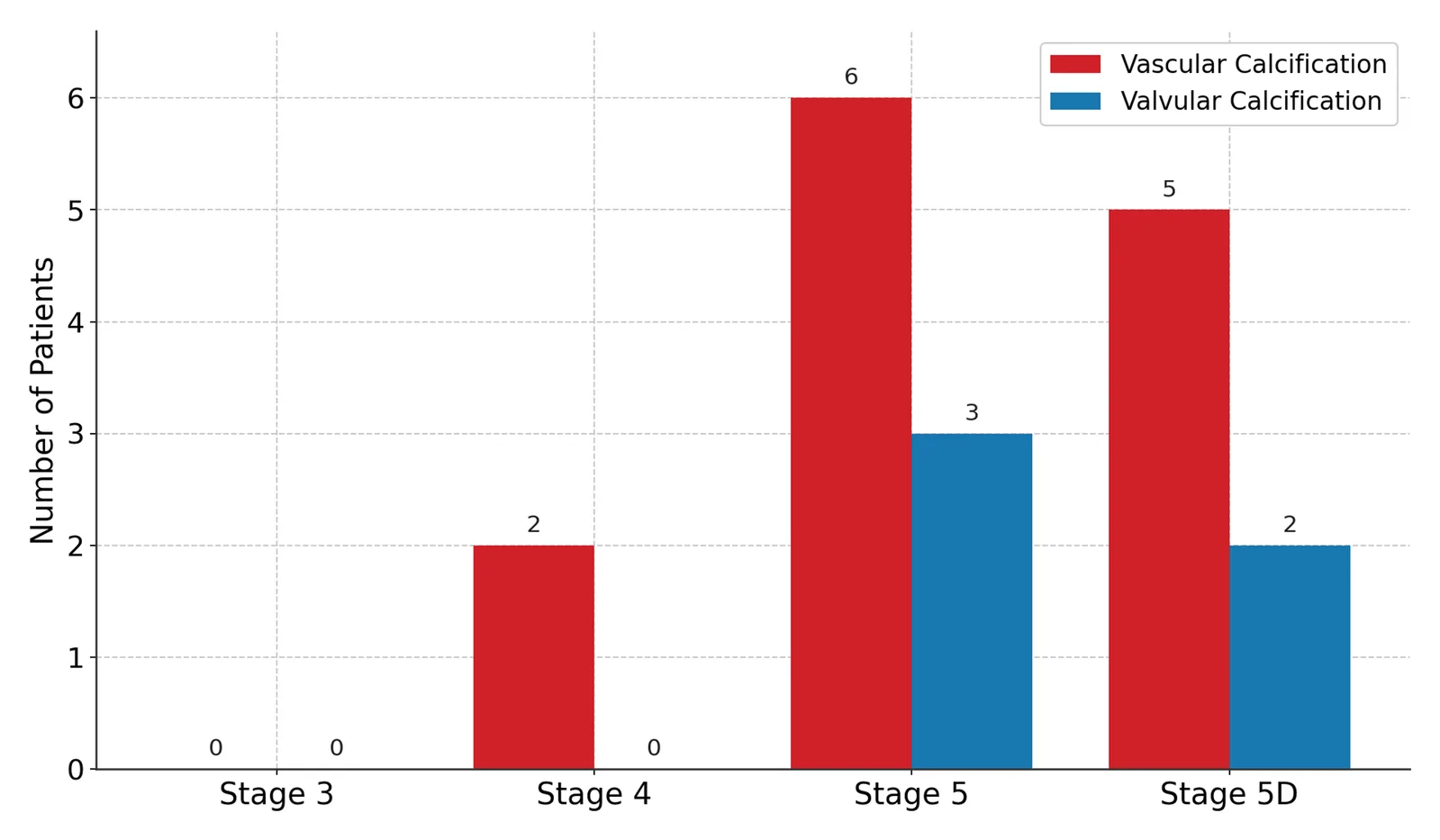
Vascular and valvular calcification across CKD stages (imaged subset, n = 57). Calcification was absent at stage 3 and clustered in stages 5 and 5D, with vascular involvement predominating over valvular involvement. CKD, chronic kidney disease

Extraskeletal calcification showed the same stage-dependent pattern. None of the patients imaged at stage 3 (*n* = 8) showed calcification of any kind. Vascular calcification increased from 2 (12.5%) at stage 4 to 6 (25.0%) at stage 5 and 5 (55.6%; 95% CI 26.7-81.1) at stage 5D, a significant trend across stages (trend χ² = 7.95, df = 1, *P* = 0.005; omnibus χ² = 8.88, df = 3, *P* = 0.031), giving an overall prevalence of 13 of 57 (22.8%; 95% CI 13.8-35.2). Valvular calcification first appeared at stage 5, in 3 (12.5%), and reached 2 (22.2%) at stage 5D, likewise showing a significant trend (χ² = 4.15, df = 1, *P* = 0.042), with an overall prevalence of 5 of 57 (8.8%; 95% CI 3.8-18.9) (Table 3; Figure 4).

**Table 3:** Vascular and valvular calcification across stages, n (%).

Stage	Imaged (*n*)	Vascular calcification, *n* (%)	Valvular calcification, *n* (%)
3	8	0 (0.0)	0 (0.0)
4	16	2 (12.5)	0 (0.0)
5	24	6 (25.0)	3 (12.5)
5D	9	5 (55.6)	2 (22.2)
Overall	57	13 (22.8)	5 (8.8)

Correlation analyses

Patients with vascular or valvular calcification displayed higher serum phosphorus and iPTH, and lower albumin-corrected calcium, than those without calcification, and alkaline phosphatase rose in step with markedly elevated iPTH (above 300 pg/mL). Vitamin D status, although widely deficient, did not differ according to calcification status (Table 4).

**Table 4:** Direction of association between biochemical parameters and the presence of vascular calcification (n = 57) Directions of association are summarised from the stage-wise biochemical and calcification data presented in Tables 2-3. Biochemical prevalences were derived from the full cohort (*n* = 150); calcification status was determined in the imaged subset (*n* = 57), comprising 13 patients with and 44 patients without vascular calcification. Alkaline phosphatase was elevated predominantly among patients with iPTH levels above 300 pg/mL. The table summarises directional relationships and does not represent a direct statistical comparison between calcification-positive and calcification-negative patients. iPTH, intact parathyroid hormone

Parameter	Calcification present (*n* = 13)	Calcification absent (*n* = 44)	Direction of association
Serum phosphorus	Higher	Lower	Higher in calcification-positive patients
Intact parathyroid hormone (iPTH)	Higher	Lower	Higher in calcification-positive patients
Albumin-corrected serum calcium	Lower	Higher	Lower in calcification-positive patients
25-hydroxyvitamin D (25(OH)D)	Similar	Similar	No difference between groups

## Discussion

This single-centre Central Indian cohort shows a coherent, stage-associated pattern of CKD-MBD. SHPT was the earliest abnormality, present in nearly half of stage 3 patients and rising to about 90% by stage 5, with a broader distribution in stage 5D that encompassed both suppressed PTH, suggestive of low bone turnover, and markedly elevated PTH. Calcium and phosphorus derangements were infrequent in stages 3-4 but became commonplace in stages 5-5D. Vitamin D deficiency was widespread (58-80%) across all stages and showed no clear stage gradient. These patterns are internally consistent and map closely onto observations from large clinical cohorts [3,14-17].

Stage-wise biochemical patterns

Our findings (an early rise in PTH from stage 3, with calcium and phosphate remaining within reference intervals until later) mirror findings from large, non-referred populations. In the Study to Evaluate Early Kidney Disease (SEEK), which enrolled approximately 1,800 patients, abnormal mineral metabolism was evident well before overt calcium and phosphate disturbance, and a substantial proportion of patients had elevated PTH once eGFR fell below 60 mL/min/1.73 m². This is highly concordant with our stage 3 signal and the progressive increase to about 90% by stage 5 [3].

At dialysis initiation, our observation that calcium was frequently near-normal while phosphate was already elevated is consistent with cohorts of incident dialysis patients, in which calcium tends to be maintained close to normal even as phosphate rises [18].

Vitamin D status

The widespread vitamin D deficiency in our cohort (58%-80%), without a clear stage gradient, reflects reports from the Kidney Early Evaluation Program (KEEP), the National Health and Nutrition Examination Survey (NHANES), and other cohorts in which deficiency is highly prevalent irrespective of CKD stage. As emphasised in the literature, assay variability and seasonal or sampling differences complicate interpretation; vitamin D values are therefore best read in conjunction with calcium-phosphate-PTH trends [16,19,20].

Alkaline phosphatase and PTH: markers of bone turnover

In our cohort, ALP was elevated predominantly in patients with iPTH >300 pg/mL, supporting its role as a biochemical marker of increased bone turnover. This accords with guideline consensus and prior work: although bone biopsy remains the diagnostic gold standard, extreme values of PTH and ALP are useful for identifying the turnover extremes that most influence management [6,8,10,21].

Vascular and valvular calcification

Vascular and valvular calcification showed a clear stage-linked increase: absent in stage 3, 12.5% (vascular) in stage 4, 25% (vascular) and 12.5% (valvular) in stage 5, and 55.6% (vascular) and 22.2% (valvular) in stage 5D. These absolute figures are lower than in many published series, largely because of the imaging modalities used. Lateral abdominal radiography for vascular calcification and echocardiography for valvular calcification are both less sensitive than computed tomography (CT). CT-based methods, including electron-beam computed tomography (EBCT) and multislice computed tomography (MSCT), detect cardiovascular calcification in 47%-83% of patients with stage 3-5 CKD and 51%-93% of dialysis patients, while echocardiographic studies report valvular calcification in 20%-47% of dialysis patients [22-24].

Despite the lower absolute prevalence in our study, the progressive stage-wise gradient we observed is fully consistent with the broader literature. Importantly, both abdominal aortic calcification on plain radiography and valvular calcium on echocardiography correlate reasonably with CT-derived coronary calcium scores, which supports the clinical validity of our findings [22,23,25].

Risk associations

In our analysis, calcification-positive patients had higher phosphorus and iPTH and lower corrected calcium, whereas vitamin D status did not differ. These associations echo the wider literature: age is the most consistent predictor, while hyperphosphataemia, high iPTH or ALP, male sex, diabetes, dialysis vintage and an elevated calcium-phosphate product have been variably linked to calcification across studies. Our observations thus fit within the biologically plausible, multifactorial risk profile recognised internationally [9,26,27].

Clinical interpretation

Taken together, these findings reinforce three practice-relevant messages: (1) monitor early and serially from stage 3; (2) act on persistently high phosphate and avoid hypercalcaemia; and (3) treat PTH trends rather than single values, reserving vitamin D analogues for severe, progressive SHPT. These messages are entirely consistent with the KDIGO 2017 recommendations [3,10].

Strengths and limitations

The strengths of this study include detailed stage-wise characterisation across CKD 3-5D, integration of biochemical and imaging data, and identification of a suppressed-PTH subgroup among dialysis patients. Several limitations should be acknowledged. The design was retrospective, cross-sectional and single-centre, so that causal inference and temporal sequence cannot be established, and the findings may not be generalisable beyond a comparable population. Radiological assessment was confined to a modest subset (*n* = 57) that was not systematically ascertained, since imaging was met by out-of-pocket expenditure, and selection bias within this sub-cohort cannot be excluded. Data on concomitant medications, including phosphate binders, active vitamin D, calcimimetics and vitamin K antagonists, were not available, so the contribution of treatment to the observed patterns - particularly the suppressed-iPTH subgroup at stage 5D - could not be assessed. No adjustment was made for potential confounders such as age, diabetes or dialysis vintage. Bone biopsy was not performed, and inferences regarding bone turnover therefore rest on biochemical markers alone. Further limitations include the use of MDRD-derived eGFR rather than the contemporary CKD-EPI equation, and reliance on radiography and echocardiography rather than CT, which probably underestimated the true prevalence of calcification. FGF-23, although mechanistically the earliest marker of disordered mineral metabolism, was not available for measurement.

## Conclusions

This Central Indian cohort demonstrates the classical stage-associated pattern of CKD-MBD: early secondary hyperparathyroidism from stage 3, with more frequent calcium-phosphate abnormalities and vascular and valvular calcification at advanced stages. The high background prevalence of vitamin D deficiency and the frequent late presentation lend a distinctive regional character to this profile. The findings support serial biochemical monitoring from stage 3 onwards, attention to modifiable factors such as hyperphosphataemia and calcium loading, and interpretation of PTH as a trend rather than a single value, to reduce cardiovascular risk in Indian practice.
